# Safety evaluation of high-risk myocardial micro-biopsy in a swine model

**DOI:** 10.1007/s00380-021-01995-9

**Published:** 2021-11-23

**Authors:** Arvin Chireh, Mikael Sandell, Rikard Grankvist, Victoria Lövljung, Jonathan al-Saadi, Fabian Arnberg, Johan Lundberg, Magnus Settergren, Staffan Holmin

**Affiliations:** 1 Department of Clinical Neuroscience, Karolinska Institutet, Stockholm, Sweden; 2 Division of Micro and Nanosystems, Royal Institute of Technology, Stockholm, Sweden; 3 MedTechLabs, Solna, Sweden; 4 Department of Neuroradiology, Karolinska University Hospital, Stockholm, Sweden; 5 Department of Cardiology, Karolinska University Hospital, Stockholm, Sweden; 6 Department of Medicine, Karolinska Institutet, Stockholm, Sweden

**Keywords:** Endomyocardial biopsy, Micro-biopsy, Safety, Interventional cardiology

## Abstract

The objective of the study was to investigate the safety profile of high-risk micro-endomyocardial biopsy (micro-EMB) compared to conventional EMB in a large animal model. Twenty pigs were subjected to a maximum of 30 consecutive biopsies, including sampling from the free ventricular wall, with either micro-EMB (*n* = 10) or conventional EMB (*n* = 10). There were no major complications in the micro-EMB group (0/10), compared to six major complications in the EMB group (6/10; *p* = 0.003). Survival analysis further highlighted these differences (*p* = 0.004). There were significantly higher volumes of pericardial effusion in the EMB group (*p* = 0.01). The study shows a safety advantage of micro-EMB compared to standard EMB in the experimental high-risk circumstances investigated in this animal study. These results indicate enhanced possibilities to collect samples from sensitive areas by using the micro-EMB technique instead of standard EMB.

**Supplementary Information:**

The online version contains supplementary material available at 10.1007/s00380-021-01995-9.

## Introduction

Endomyocardial biopsy (EMB) is an established method to obtain cardiac biopsies through the endovascular route. The method is primarily used to detect allograft rejection (after cardiac transplantation) and for investigating selected cases of unexplained heart failure [1, 2]. Despite the complication risks and a relatively low sensitivity, EMB is considered the reference method for diagnosis of several cardiac diseases, such as myocarditis [2, 3]. Studies report a variable complication rate between less than 1% and 8.9%, with cardiac tamponade and arrhythmias among the severe complications [4–7]. Despite agreement that bi-ventricular biopsies could improve sensitivity, left ventricle EMB (LV-EMB) is not widely considered safe, although recent data suggest relatively low risks when performed by experienced operators [6, 8, 9].

To improve the safety and value of EMB, we previously developed a novel EMB device and analysis protocol, referred to as micro-EMB [10]. The novel method features a significantly miniaturized and flexible biopsy device coupled to low-input analysis methods such as RNA-sequencing. Our previous studies of LV-micro-EMB in swine showed favorable safety outcomes as well as other outcomes such as navigability, sample quality, and ability to detect disease related changes [10, 11]. For instance, it was shown that each micro-EMB attempt caused an injured area of 0.3 mm^2^ in the endomyocardium, compared to 18 mm^2^ with a conventional device [10]. Although this minimized trauma suggests lower procedural risks, a dedicated safety study was not performed. Therefore, this study was conducted to investigate the safety profile of micro-EMB compared to standard EMB in a large animal model.

We hypothesized that micro-EMB will cause fewer major complications compared to conventional EMB. However, since severe complications from EMB are relatively rare (although clinically significant), an impractical number of animals would be required to detect potential differences between micro-EMB and conventional EMB with standard clinical protocols. Therefore, we further hypothesized that an aggressive sampling approach, including sampling from the free wall, would increase the number of events sufficiently to enable statistical analysis despite a relatively small number of animals.

To this end, we designed this study to compare the safety profiles of micro-EMB and standard EMB in a limited number of animals, using a high-risk sampling approach.

## Materials and methods

### Experimental design

**Fig. 1:**
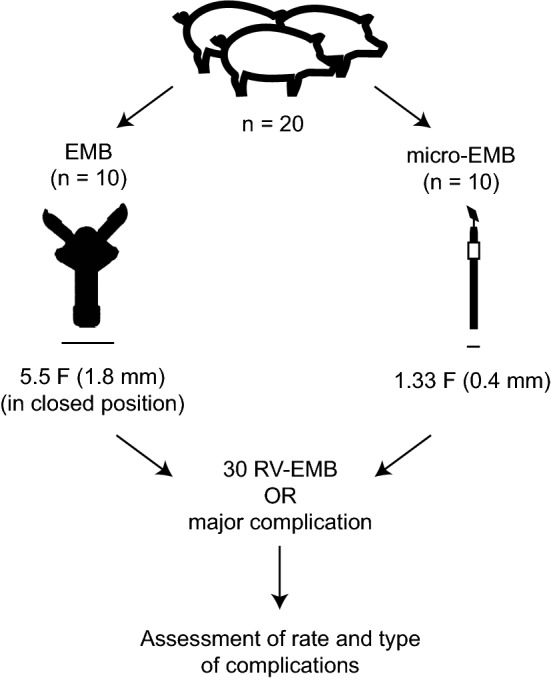
Study design. Twenty pigs were subjected to either micro-EMB with a 0.4 mm micro-EMB device (*n* = 10) or EMB with a standard 5.5 F Cordis Biopsy Forceps (*n* = 10). A high-risk sampling schedule was employed, including a maximum of 30 biopsies taken from various parts of the right ventricle, including the free wall. The experiment was terminated either upon a major complication or 30 biopsies without any major complication. Acute complications were assessed and compared on group level

Twenty mixed breed Yorkshire-Swedish farm pigs (Table 1, Online Resource 1) were subjected to either micro-EMB (*n* = 10) or EMB (*n* = 10; Fig. 1), using a high-risk sampling approach. The animals were continuously monitored in the operating room, and transthoracic echocardiography (TTE) was performed after each biopsy attempt. A maximum of 30 biopsies were taken from various parts of the right ventricle, including the free wall. The experiment was terminated either upon a major complication or a maximum of 30 biopsies without any major complication. A major complication was defined as a cardiac event (such as arrhythmia or pericardial effusion) causing death or sustained hemodynamic compromise (mean artery pressure (MAP) below 45 for at least 20 min). Hemodynamic compromise due to pericardial effusion was classified as tamponade. The frequencies and character of the severe complications were compared on group level between the two groups.

**Table 1:** Animal characteristics and outcome

Group	Gender (*n* female/total)	Weight (median and range, kg)	Outcome (*n* events/total)
Micro-EMB	7/10	39.9 (36.1–45.0)	0/10
Conventional EMB	7/10	40.4 (32.5–48.3)	6/10

### Ethical considerations

The study was conducted in accordance with national and local guidelines for Sweden and Karolinska Institutet, respectively. All animal experiments were approved from the local ethics committee (Stockholms Norra Djurförsöksetiska Nämnd, Stockholm, Sweden) prior to starting the experiments. All experiments were performed in accordance with ‘Principles of Laboratory Animal Care’ formulated by the National Society for Medical Research as well as the ‘Guide for the Care and Use of Laboratory Animals’ prepared by the Institute of Laboratory Animal Resources.

### Animal experiments

The animals were allocated to each study group in an alternating order, so that approximately every other animal was allocated to the micro-EMB group and the other to the standard EMB group. There was no blinding of the operator or the rest of the staff. There were no predetermined inclusion or exclusion criteria.

All animals were anesthetized and monitored using the same protocol as previously reported [10]. Throughout the intervention, the animals were monitored for complications using continuous ECG, invasive blood pressure measurement, oxygen saturation, temperature, and urine production. Animals were treated with 75 mg amiodarone intravenously (iv) as a single dose prior to cardiac catheterization and 5000 units of heparin per hour during the procedure.

Vascular access was gained by surgical cut-down of the left or right external jugular vein followed by catheterization (using Seldinger technique). An 8.5 French (F) Agilis™ NxT steerable introducer (Abbott Vascular, Menlo Park, California, USA) was inserted and advanced to the RV. The location was confirmed with ventriculography and pressure measurements through the introducer sheath. Micro-EMB samples were harvested from the myocardium using a similar technique as previously described, with two minor modifications [10]. First, the tip of the device was reduced to a total penetration depth of 2 mm (due to the reduced wall thickness of RV compared to LV). Second, the device was housed inside a guiding catheter (5F Envoy^®^ with multi-purpose D tip, Codman Neuro, Raynham, Massachusetts, United States) instead of a microcatheter since there was already an introducer sheath placed in the RV. Conventional EMB samples were taken with a 5.5 F Cordis Biopsy Forceps (Cordis, Hialeah, Florida, United States), using standard clinical technique supervised by a consultant in interventional cardiology. All biopsies were initially directed towards the right interventricular septum followed by incremental variations to the sampling location. The bioptome was aimed towards the inferior, middle, and superior portions of the septum, as well as the anterior, lateral, and posterior free wall.

After each micro-EMB attempt, the device was assessed under light microscopy to ensure that a biopsy sample was taken. Cases with none or insufficient sample material were considered as failed biopsy attempts and were not counted as performed biopsies. After each biopsy (regardless of method), a quick echocardiography examination was performed to detect pericardial effusion (Px). A Philips ultrasound machine (CX 50) with an S5-1 cardiac probe was used, placing the probe in a left midaxillary position, aiming for a view resembling a human short-axis view.

When the endpoint was reached (major complication or 30 biopsies), the animal was euthanized with a lethal dose of pentobarbital. Sternotomy was performed to evaluate pericardial fluid and the heart was extracted for gross inspection.

### Data analysis and statistics

All animals (*n* = 20) were included in the analysis. Descriptive statistics were used to assess distribution of weight and gender between the groups. Values are reported in median and range unless otherwise specified.

Chi-square test was used to test the overall difference between the groups in terms of complication frequencies. Kaplan–Meier survival analysis was performed using the “survival” package (version 3.2.3) in R. Major complications were classified as events. Animals were censored after 30 successful biopsy attempts. Log-rank test was used to determine statistical difference between the two groups in the survival analysis. Unpaired *t* test was used to test the difference of pericardial effusion between the groups.

All statistical analyses were performed using the R statistical computing language, version 4.0.2. *p* values of 0.05 or less were considered significant.

## Results

**Fig. 2:**
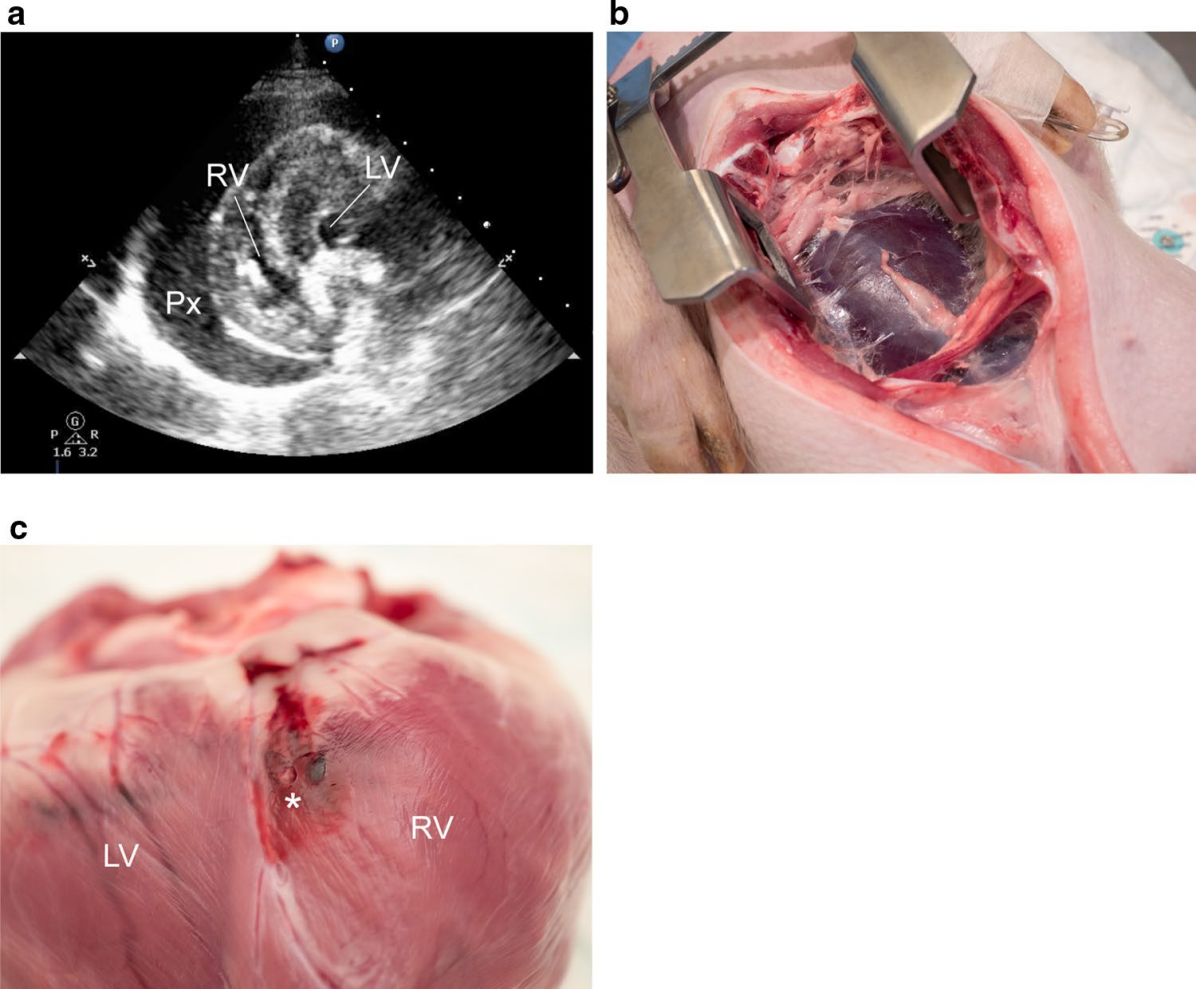
Tamponade in an animal subjected to high-risk conventional EMB. **a** Transthoracic echocardiogram showed extensive pericardial effusion after 11 biopsies, while the animal was hypotensive. **b** Sternotomy after euthanasia revealed a blood-filled pericardial sac, measuring 180 ml. **c** Closer inspection of the heart showed hematomas on the posterior surface of the right ventricle (RV), with a perforation (asterisk)

Twenty swine (Table 1) were subjected to extensive biopsy sampling with either micro-EMB (*n* = 10) or EMB (*n* = 10), including sampling from the free ventricular wall. In the micro-EMB group, 300 of 325 (92%) biopsy attempts were successful, compared to 185 of 189 (98%) in the EMB group. Failed biopsy attempts were not counted as performed biopsies. The number of major complications was 0/10 (0%) in the micro-EMB group and 6/10 (60%) in the conventional EMB group, which was significantly different between the groups (*p* = 0.003). All major complications were in the form of acute cardiac tamponade characterized by hypotension and pericardial effusion (Fig. 2).

**Fig. 3:**
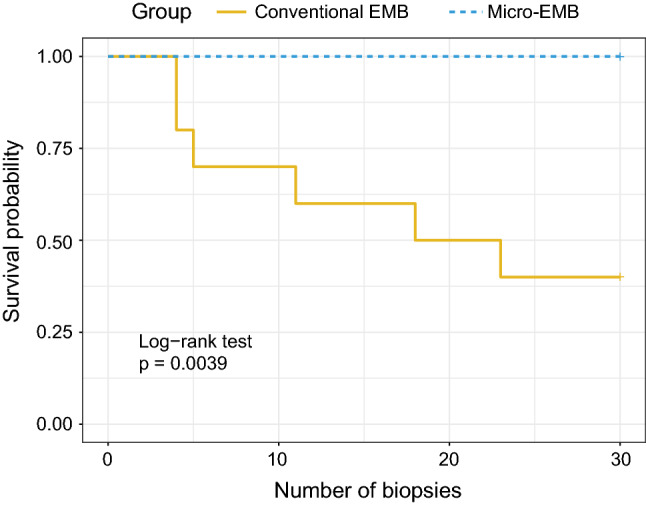
Kaplan–Meier survival analysis. Survival plot shows a significant difference between micro-EMB and conventional EMB (*p* = 0.004) after high-risk biopsy sampling in swine (*n* = 20). Major complications such as severe arrhythmia or acute tamponade were considered as events. Animals were censored after 30 biopsies

Survival analysis was performed to investigate time-to-event (Fig. 3). Major complications, such as severe arrhythmia and tamponade, were considered as events, although only tamponade occurred. Animals were censored after 30 biopsies. The results showed significant survival differences between the groups (*p* = 0.004, log-rank test). There were no events in the micro-EMB group, while the conventional group suffered from evenly distributed events.

**Fig. 4:**
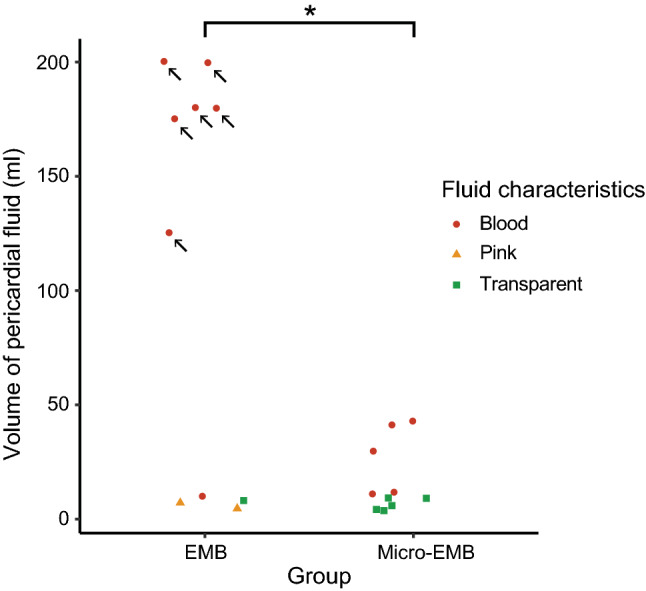
Volume and characteristics of pericardial fluid post-mortem. Following the series of high-risk biopsy sampling (*n* = 20 animals), there were higher volumes of pericardial effusion (blood) in the EMB group compared to micro-EMB (*p* = 0.01). Animals indicated with arrows (*n* = 6) suffered from tamponade whereas the others were hemodynamically stable

After euthanasia, the volume and appearance of the pericardial fluid was assessed (Fig. 4). Blood-colored pericardial effusion was detected in 12 animals, of which seven were from the EMB group (58%). Half of these animals (*n* = 6) had a known tamponade, with pericardial effusion ranging between 125 and 200 ml, whereas the others (*n* = 6) had an undetected perforation with a blood-colored effusion ranging between 10 and 43 ml. Overall, the volume of pericardial fluid was significantly higher in the conventional EMB group compared to the micro-EMB group (*p* = 0.01).

**Fig. 5:**
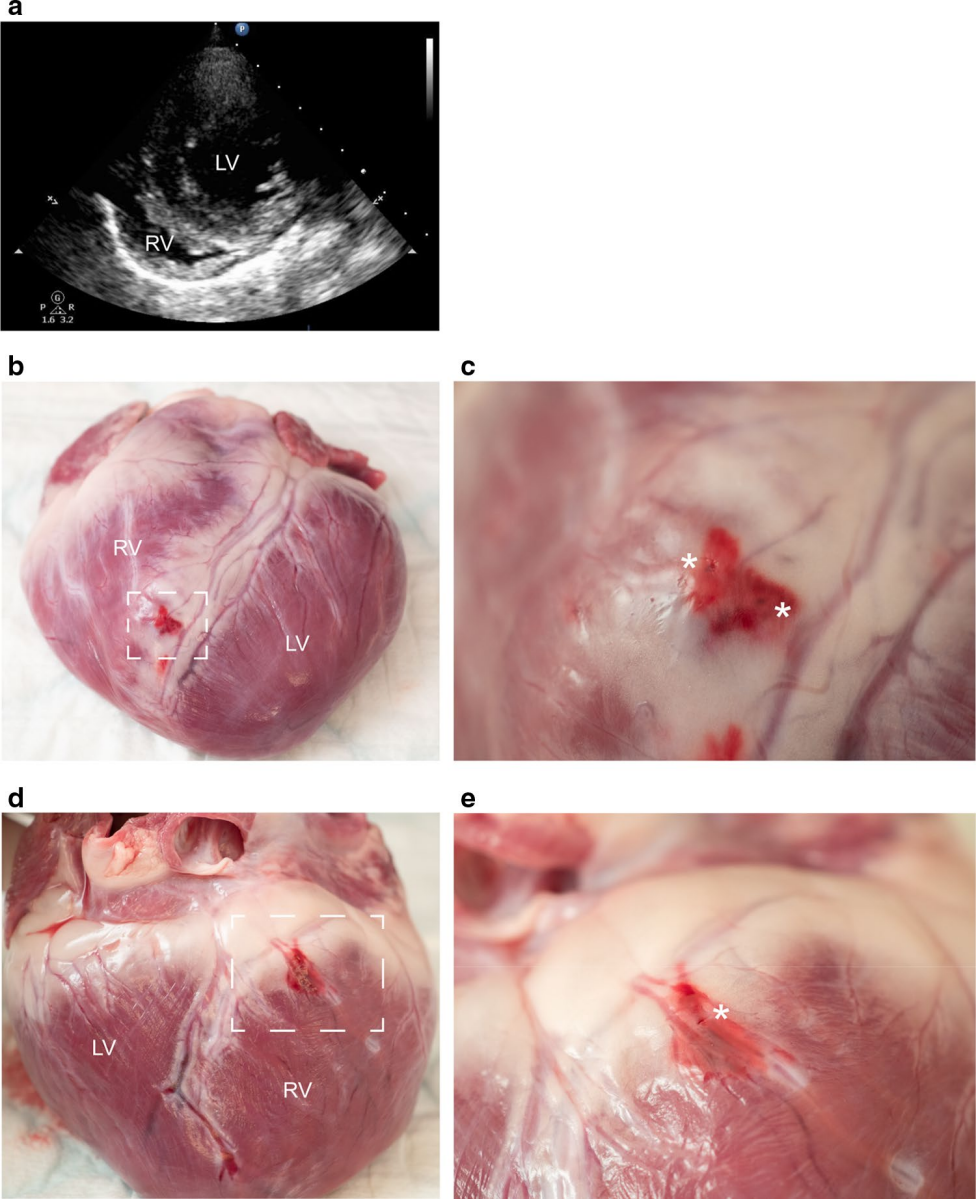
Echocardiography and post-mortem analysis in one animal subjected to high-risk micro-EMB (30 biopsies). This animal was hemodynamically stable and had no detectable px on TTE (**a**), but post-mortem analysis revealed 11 ml of blood-colored pericardial effusion. **b** Anterior wall of the RV shows hematoma. **c** Close-up of dashed area in (**b**) reveals two suspected perforations (asterisks). **d** The posterior wall of the RV also shows a hematoma with one suspected perforation (**e**, asterisk)

To further investigate the timing and location of the cardiac wall perforations, post-mortem tissues and transthoracic echocardiogram (TTE) examinations were reviewed. Post-mortem analysis of animals with pericardial blood (*n* = 12) revealed hematomas and suspected perforations on the anterior (*n* = 2), posterior (*n* = 7), or multiple (*n* = 3) aspects of the myocardial wall (Online Resource 1). On TTE, pericardial effusion was detected in all animals with tamponade but not in the non-tamponade animals, impeding the possibility to determine the time (number of biopsies) to cardiac wall perforation in those cases. Examples of a negative TTE examination and post-mortem hematoma in one animal subjected to micro-EMB are shown (Fig. 5).

To assess rates of arrhythmia, the animals were continuously monitored with ECG. All animals (in both groups) exhibited temporary tachycardia when penetrating the myocardium with the biopsy devices, but no animal suffered from prolonged arrhythmia with hemodynamic compromise.

**Fig. 6:**
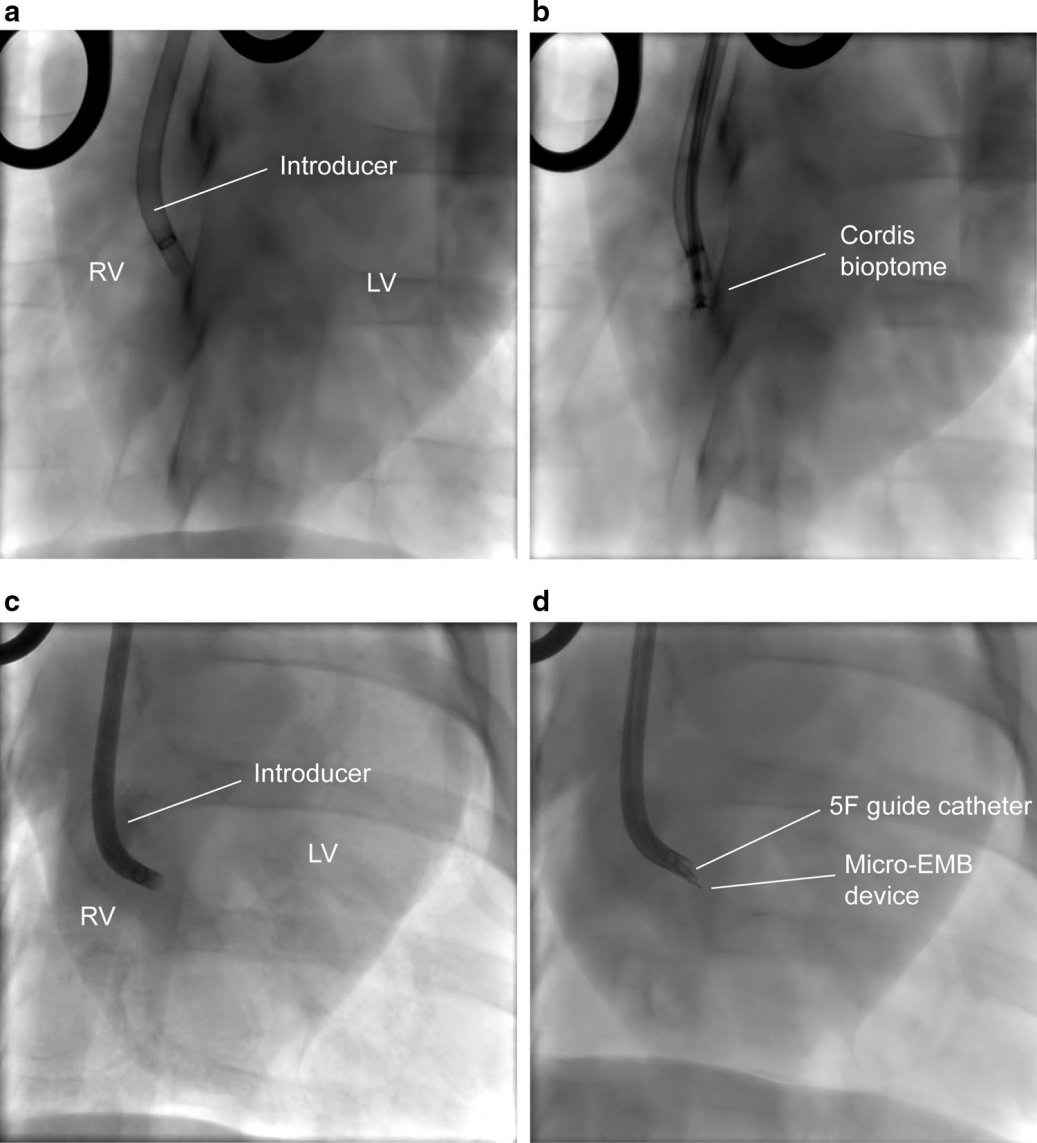
Fluoroscopy images in PA projection, showing navigability differences between EMB and micro-EMB. **a**, **b** Shows how the flexible introducer sheath (8.5 F) changes angle after the conventional EMB bioptome is advanced (**b**). **c**, **d** Shows intact curvature of the introducer after advancing the 5F guide catheter housing the micro-EMB device (**d**). The same introducer sheath was used (8.5 F), although with a slightly different curve. In **c** and **d**, the introducer is filled with contrast medium due to preceding ventriculography

As part of the study design, sampling location was varied after every five biopsies. Incidentally, it was noted that the conventional EMB device was difficult to accurately navigate due to stiffness and size of the bioptome (Fig. 6a, b). In comparison, micro-EMB was easier to direct and also featured a guide catheter with a bent tip to allow another dimension of movement (Fig. 6c, d).

## Discussion

Micro-EMB was recently developed as a potential alternative to conventional EMB, aiming to increase flexibility and safety. In this study, we compared the safety profiles of micro-EMB and conventional EMB in a limited number of swine, using an experimental high-risk sampling approach. As hypothesized, there was a significantly lower number of major complications in the micro-EMB group compared to conventional EMB in these circumstances, despite the few animals enrolled. In the micro-EMB group, there was no major complication, while there was a tamponade in six of ten animals in the conventional EMB group. These results clearly suggest a safety advantage of micro-EMB when performing extensive sampling from areas of the right ventricle that are prone to complications.

The high complication rate in the EMB group might appear surprising compared to available clinical data, which suggest a major complication rate of less than 1% [12]. However, as mentioned in the introduction, the high-risk sampling approach employed in this study was different from a standard clinical EMB procedure. We performed biopsies repeatedly from several regions of the myocardial wall, including the right ventricular free wall, which was expected to yield a higher complication frequency. This approach was selected to highlight potential differences between the methods without the need of a large number of animals. Despite the discrepancy between clinical protocols and the high-risk approach used in this study, the comparison is still relevant for a number of reasons. For instance, there are diseases with patchy distributions, or tumors, that cannot be detected by exclusively targeting the septum and can therefore require sampling from sensitive areas [8, 13, 14]. Moreover, repeated surveillance biopsies after cardiac transplantation can cause extensive fibrosis, making it increasingly difficult to obtain adequate samples without varying the sampling location [15, 16]. Overall, since inadequate sampling is one of the main limitations of EMB [14, 17], the suggested safety advantage of micro-EMB in high-risk scenarios has the potential to widen the diagnostic utility compared to standard EMB.

Post-mortem analysis revealed pericardial effusion (blood) and macroscopic signs of perforation in animals from both groups. However, hemodynamic compromise did not occur in the micro-EMB group. Since these smaller effusions were only revealed post-mortem, the timing of the perforation could not be established. Given the high number of biopsies from varied locations, perforation at some point should not come as a surprise. This finding highlights that caution and appropriate monitoring is warranted regardless of device, but that the risk of perforation causing hemodynamic compromise was significantly higher with the conventional method in the studied circumstances.

Although arrhythmia is one of the feared complications of EMB, no severe arrhythmia with hemodynamic compromise occurred during the study. These results should be interpreted with caution since the animals were given anti-arrhythmic drugs, due to known myocardial sensitivity in pig, as well as lack of follow-up.

Extensive handling of both micro-EMB and EMB devices revealed a flexibility advantage of micro-EMB, enabling more accurate sampling. The conventional EMB device is relatively difficult to navigate accurately due to size and stiffness, even when using a flexible introducer sheath. Although crude positional changes were still possible, the steering difficulties could be relevant in scenarios where an accurate targeting is needed, such as in patchy disease or extensive fibrosis. As discussed in our previous study, micro-EMB could potentially be used in conjunction with interventional MRI guidance, or other navigational tools, to refine accuracy even further [11].

The study design was chosen to compare acute complication frequencies in a few animals and without the need of a longitudinal study. A maximum of 30 biopsies, including sampling from the free ventricular wall, was performed to ensure adequate statistical power while reducing the required number of animals. As a result, only acute and major complications such as cardiac wall perforation and acute arrhythmias were evaluated. Although clinically relevant, long-term complications such as valve dysfunction or myocardial scarring were not assessed. Complications related to the vascular access and non-cardiac complications such as pneumothorax or stroke were also not considered. Since the aim of the study was to compare complication frequencies (in a high-risk scenario), not to estimate mortality risks, tamponade was considered an endpoint and no attempt was made to treat the animals (e.g., by pericardiocentesis). Moreover, the study was performed in an animal model and the sampling scheme was vastly different from standard clinical protocols. Consequently, the results cannot be used to estimate risks in a standard clinical scenario, where concurrent EMB has a significantly lower rate of major complications compared to the numbers presented in this study. Since the biopsy location was also varied, the complication risks from specific biopsy locations could not be evaluated.

It should also be noted that the diagnostic utility of micro-EMB depends on advances in molecular-based methods for diagnosis, since the samples are too small for conventional histology. In previous studies, we showed the capabilities to generate high quality RNA data from swine micro-biopsy samples, as well as detecting tissue changes after myocardial infarction [10, 11]. Moreover, Halloran and colleagues have developed and explored a gene expression profile test for allograft rejection, based on standard EMB samples [18]. The clinical utility of these gene expression tests, particularly from micro-biopsy samples, are yet to be investigated in clinical trials. Although these considerations limit the immediate translational outlook of the study, the safety comparison between micro-EMB and standard EMB is still relevant since micro-EMB is intended as a future alternative to EMB (within and beyond the current clinical indications).

Another important limitation of the study is the lack of blinding, which causes inevitable bias. Since the handling and appearance of the devices are fundamentally different, it was not possible to blind the operator. Nevertheless, all biopsies were performed by the same operator to avoid issues related to operator experience.

In conclusion, this study shows a safety advantage of micro-EMB compared to EMB in the employed animal model, consisting of high-risk sampling from several areas, including the free ventricular wall. This suggested safety advantage in high-risk scenarios has the potential to widen the diagnostic utility of myocardial biopsies in cases where molecular-based tests can replace or complement histology. Future studies should focus on long-term safety of biopsies from specific targets, including non-acute complications such as valve injuries. The sampling protocols should also be closer to real-world clinical scenarios, and preferably using a clinical grade micro-EMB device. Future studies should also assess the diagnostic capabilities of micro-EMB, as discussed elsewhere [11].

## Supplementary Information

Below is the link to the electronic supplementary material.

Supplementary file1 (PDF 45 KB)

## Abbreviations

EMB: Endomyocardial biopsy
F: French
TTE: Transthoracic echocardiography
LV: Left ventricle
MAP: Mean artery pressure
PA: Posteroanterior
Px: Pericardial effusion
RV: Right ventricle

## Declarations

### Animal studies

The work had prior ethical approval from the local ethics committee (Stockholms Norra Djurförsöksetiska Nämnd, Stockholm, Sweden) and was conducted in accordance with local and national ethical guidelines, as well as the ARRIVE guidelines.
