# Supplementary material for: Safety evaluation of high-risk myocardial micro-biopsy in a swine model
Source: Heart Vessels. 2021 Nov 23;37(4):697–704. doi: 10.1007/s00380-021-01995-9 (PMC8917023; doi:10.1007/s00380-021-01995-9)
Supplement: Supplementary file 1 — Supplementary file1 (PDF 45 KB) [file 380_2021_1995_MOESM1_ESM.pdf]

Title: Safety evaluation of high-risk myocardial micro biopsy in a swine model

Journal: Heart and Vessels

Authors: Arvin Chireh. Mikael Sandell. Rikard Grankvist. Victoria Lövljung. Jonathan al-Saadi. Fabian Arnberg. Johan Lundberg. Magnus Settergren. Staffan Holmin.

Corresponding author: staffan.holmin@ki.se. Department of Clinical Neuroscience, Karolinska Institutet, Stockholm, Sweden.

ID	Sex	Weight (kg)	Group	Number of biopsies	Tamponade	Volume blood (ml)	Hematoma	Location hematoma	Effusion on TTE
1	m	41	EMB	4	Yes	180	Yes	anterior	Yes
2	m	39,8	EMB	30	No	0	Yes	anterior	No
3	f	47,1	EMB	23	Yes	200	Yes	anterior	Yes
4	f	48,3	EMB	30	No	0	Yes	anterior	No
5	f	40	Micro-EMB	30	No	30	Yes	posterior	No
6	f	41,3	Micro-EMB	30	No	0	Yes	posterior	No
7	m	39,7	Micro-EMB	30	No	12	Yes	posterior	No
8	m	38	Micro-EMB	30	No	0	No		No
9	m	40,9	EMB	18	Yes	200	Yes	posterior, apical	Yes
10	m	42,4	Micro-EMB	30	No	0	No		No
11	f	39	Micro-EMB	30	No	0	No		No
12	f	38	EMB	30	No	0	Yes	posterior	No
13	f	45	Micro-EMB	30	No	43	Yes	posterior	No
14	f	41,5	Micro-EMB	30	No	0	Yes	anterior	No
15	f	37,3	Micro-EMB	30	No	41	Yes	posterior	No
16	f	36,1	Micro-EMB	30	No	11	Yes	anterior, posterior	No
17	f	42,1	EMB	30	No	10	Yes	posterior, apical	No
18	f	35,9	EMB	4	Yes	175	Yes	posterior	Yes
19	f	34,6	EMB	11	Yes	180	Yes	posterior	Yes
20	f	32,5	EMB	5	Yes	125	Yes	posterior	Yes
